# MRI annotation using an inversion-based preprocessing for CT model adaptation

**DOI:** 10.1186/s41747-025-00626-6

**Published:** 2025-09-19

**Authors:** Hartmut Häntze, Lina Xu, Maximilian Nikolas Rattunde, Leonhard Donle, Felix J. Dorfner, Alessa Hering, Jawed Nawabi, Lisa C. Adams, Keno K. Bressem

**Affiliations:** 1 Department of Radiology, Charité—Universitätsmedizin Berlin corporate member of Freie Universität Berlin and Humboldt Universität zu Berlin, Berlin, Germany; 2 Diagnostic Image Analysis Group, Radboud University Medical Center, Nijmegen, GA The Netherlands; 3 Athinoula A. Martinos Center for Biomedical Imaging, Massachusetts General Hospital and Harvard Medical School, Charlestown, MA USA; 4 Department of Neuroradiology, Charité—Universitätsmedizin Berlin corporate member of Freie Universität Berlin and Humboldt Universität zu Berlin, Berlin, Germany; 5 Department of Diagnostic and Interventional Radiology, School of Medicine and Health, Klinikum rechts der Isar, TUM University Hospital, Technical University Munich, Munich, Germany; 6 Department of Cardiovascular Radiology and Nuclear Medicine, School of Medicine and Health, German Heart Center, TUM University Hospital, Technical University of Munich, Munich, Germany

**Keywords:** Artificial Intelligence, Carcinoma (renal cell), Image processing (computer-assisted), Magnetic resonance imaging, Tomography (x-ray computed)

## Abstract

**Background:**

Annotating new classes in MRI images is time-consuming. Refining presegmented structures can accelerate this process. Many target classes lacking in MRI are supported by computed tomography (CT) models, but translating MRI to synthetic CT images is challenging. We demonstrate that CT segmentation models can create accurate MRI presegmentations, with or without image inversion.

**Materials and methods:**

We retrospectively investigated the performance of two CT-trained models on MRI images: a general multiclass model (TotalSegmentator); and a specialized renal tumor model trained in-house. Both models were applied to 100 T1-weighted (T1w) and 100 T2-weighted fat-saturated (T2wfs) MRI sequences from 100 patients (50 male). Segmentation quality was evaluated on both raw and intensity-inverted sequences using Dice similarity coefficients (DSC), with reference annotations comprising manual kidney tumor annotations and automatically generated segmentations for 24 abdominal structures.

**Results:**

Segmentation quality varied by MRI sequence and anatomical structure. Both models accurately segmented kidneys in T2wfs sequences without preprocessing (TotalSegmentator DSC 0.60), but TotalSegmentator failed to segment blood vessels and muscles. In T1w sequences, intensity inversion significantly improved TotalSegmentator performance, increasing the mean DSC across 24 structures from 0.04 to 0.56 (*p* < 0.001). Kidney tumor segmentation demonstrated poor performance in T2wfs sequences regardless of preprocessing. In T1w sequences, inversion improved tumor segmentation DSC from 0.04 to 0.42 (*p* < 0.001).

**Conclusion:**

CT-trained models can generalize to MRI when supported by image augmentation. Inversion preprocessing enabled segmentation of renal cell carcinoma in T1w MRI using a CT-trained model. CT models might be transferable to the MRI domain.

**Relevance statement:**

CT-trained artificial intelligence models can be adapted for MRI segmentation using simple preprocessing, potentially reducing manual annotation efforts and accelerating the development of AI-assisted tools for MRI analysis in research and future clinical practice.

**Key Points:**

- CT segmentation models can create presegmentations for many structures in MRI scans.
- T1w MRI scans require an additional inversion step before segmenting with a CT model.
- Results were consistent for a large multiclass model (*i.e.*, TotalSegmentator) and a smaller model for renal cell carcinoma.

**Graphical Abstract:**

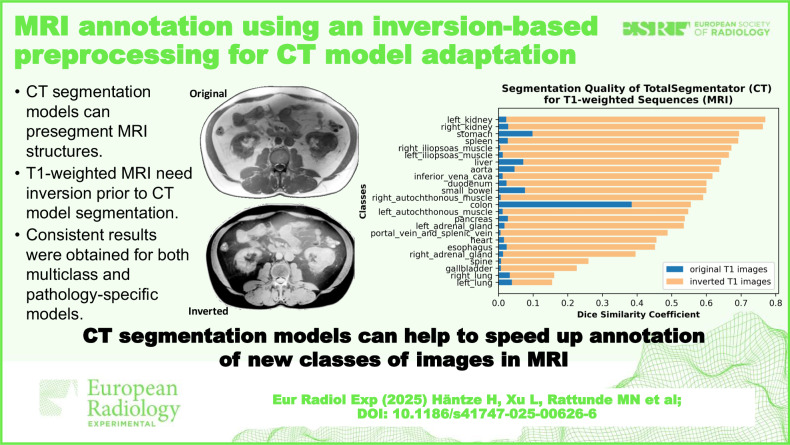

## Background

Medical image segmentation plays an important role in many automated image analysis tools. It has been well established for computed tomography (CT) scans, with multiple open-source models [1, 2] and challenges available. Segmentation of MRI scans, particularly multiorgan segmentation, has long been lacking behind, but recently, MRI models have been published that close this gap [3–6]. Nonetheless, differences between MRI and CT segmentation remain. For CT, TotalSegmentator [2] can parallelly infer 117 structures, while the MRI segmentation model, with the largest number of classes [4], is limited to 80 structures. Segmentation of pathological lesions in MRI studies has predominantly been focused on the brain [7] while segmentation of lesions in the abdominal region remains comparatively underexplored. For instance, most studies for renal tumors focus on the segmentation of CT scans [8, 9], supported by public challenges such as KiTS23 [10]. In contrast, there is a notable lack of segmentation models addressing renal pathology in MRI. Although CT is the preferred modality for diagnosing suspected renal conditions, MRI remains widely utilized and is recommended by clinical guidelines [11, 12]. This underscores the critical need for developing new segmentation approaches tailored to MRI.

Training new MRI segmentation models requires a large number of annotated images, and the more classes involved, the greater the annotation effort needed. Voxel-wise annotation can be very time-consuming, especially if the segmentations have to be created from scratch. However, if annotators instead focus on refining pre-segmented structures, *i.e*., segmentations that are not perfect but cover a large part of the target structure, the annotation process can be greatly accelerated.

Presegmentations can be created with the help of interactive annotation frameworks [13, 14] in which deep learning models try to segment target structures marked by user clicks. Alternatively, if annotations of the target structure exist for another modality (*e.g*., CT), it is possible to use those scans to retrain a new segmentation model for the target domain. For example, by coregistering corresponding CT and MRI scans [15] or by using augmented CT scans [16]. Nonetheless, implementing and training an augmented model to create pre-segmentations can be resource-intensive, time-consuming, and technically challenging. Additionally, it requires having data from both the source and target modality. Interactive annotation frameworks, on the other hand, require long-term maintenance and updates, which are not common for scientific software.

In this article, we propose to use pure CT models paired with image augmentation for the task of initial pre-segmentation creation. One key difference between MRI and CT images is that dense tissue, such as bones, appears bright (hyperdense) in CT scans but dark (hypointense) in MRI images. We attempt to minimize this difference by using negatives of MRI images and analyze whether it influences the semantic segmentation performance of models trained solely on CT data. We investigate the effects on two models: first, a general multiclass segmentation model, *i.e*., TotalSegmentator, to analyze a broad range of structures for which we can readily obtain a reference standard; second, a specialized model focused on the segmentation of renal tumors, reflecting our belief that pathological models represent a key direction for future advancements in segmentation research.

## Materials and methods

This study was approved by the local ethics committee (EA4/062/20). Due to the retrospective nature of the study, patient consent was waived.

An in-house database was scanned for patients with histopathologically-confirmed clear cell renal cell carcinoma, and pre-operative CT and MRI were extracted. Scans of patients who underwent both modalities were excluded to reduce bias. The CT data consisted of 1,012 scans from 529 patients (380 men). Average patient age was 63.3 ± 11.1 years (mean ± standard deviation). It includes early venous (*n* = 425), delayed venous (*n* = 121), arterial (*n* = 300), and non-contrast phase (*n* = 166). For the MRI data, patients were filtered to include those with both T1- and T2-weighted MRI images, resulting in 338 patients. From this group, 100 patients (50 males and 50 females) were randomly sampled, aged 61.5 ± 10.7 years (mean ± standard deviation). CT scans and MRI data were acquired using scanners from Siemens, Philips, General Electric, and Toshiba. CT scans had slice thicknesses ranging from 0.5 to 10 mm and pixel spacings between 0.56 × 0.56 mm up to 1.0 × 1.0 mm. MRI was performed at 1.5 T and 3.0 T, with slice thickness between 2.7 mm and 10 mm and pixel spacings between 0.57 × 0.57 mm up to 1.95 × 1.95 mm. All CT and MRI volumes were resampled to an isotropic voxel size of 1 × 1 × 1 mm.

A reference standard of 24 abdominal structures was created in our MR images using an automatic segmentation model [3]. For the tumor reference standard in both CT and MRI, a medical student (MR) annotated primary tumors and the corresponding kidneys. Two radiologists (L.C.A. and K.K.B.), with 5 years and 4 years of experience, respectively, then reviewed and corrected the annotations by consensus. The remaining tumor-free kidneys were segmented using the automatic model described by Häntze et al [3]. Tumors in the MRI data had a median volume of 29 cm^3^ (ranging from 0.7 cm^3^ to 1,971 cm^3^). In 45 cases, the primary tumor was located on the left side, and in 55 cases on the right side.

Two CT segmentation models were investigated. One model (*i.e*., TotalSegmentator) is external and publicly available. We used the fast version including its pretrained weights (version 2.2.1). The second model is a specialized renal tumor model, which we trained on our CT data using an nnU-Net [17]. It predicts the left and right kidneys and the primary tumor. The trained model achieved a Dice similarity coefficient (DSC) of 0.73 for tumors, 0.89 for the non-tumor kidney area, and 0.91 for the tumor-free kidney on the CT validation folds.

Both CT models were used for inference on MRI. As preprocessing, all intensities of the MRI images were first clipped to a value range between 0 and 3,000. Subsequently, negatives were created within their original intensity range. Lastly, all intensities within the first percentile were set to zero (Eq. 1).

1 $${INV}\left(x\right)=\left\{\begin{array}{c}0 \qquad \qquad {{{\rm{x}}}}\in \,{{percentile}}_{1}(X)\\ \max \left(X\right)-x+\min \left(X\right) \quad \;{else}\hfill\end{array}\right.$$

**Fig. 1:**
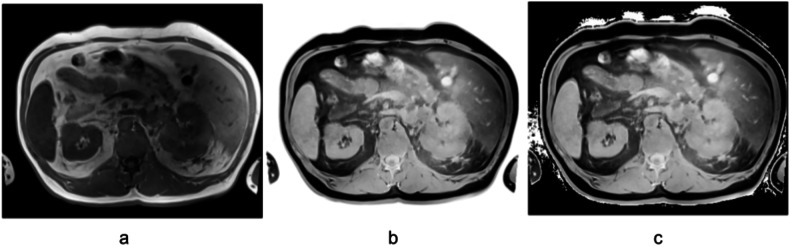
**a** Unprocessed T1-weighted image, (**b**) inverted image, and (**c**) inverted + black background image

This step ensured that the surrounding area around the patient remained black. Although this process produced some artifacts in the air-filled lungs, it proved to be very stable within the abdominal region. We then ran the TotalSegmentator-fast and the renal tumor model on both the original MRI images (Fig. 1a) and their inverted versions (Fig. 1b, c).

The models’ outputs were compared to the reference standard labels, and the DSC was calculated. Finally, the role of tumor volume was investigated, specifically in the segmentation of inverted T1-weighted (T1w) MRI. To assess significance, the Shapiro–Wilk test was used to assess normality, followed by Wilcoxon signed-rank tests and Spearman’s rank correlation, with *p*-values corrected using the Benjamini–Hochberg procedure.

## Results

Without preprocessing, TotalSegmentator failed to detect any classes in the T1w sequences, except for the colon (DSC 0.38). For the T2-weighted fat-saturated (T2wfs) sequences, TotalSegmentator could segment eight large abdominal organs with DSC values above 0.40 (right kidney 0.60; spleen 0.55; small bowel 0.55), but struggled to segment blood vessels (aorta 0.17) and muscles (right iliopsoas muscle 0.19).

**Fig. 2:**
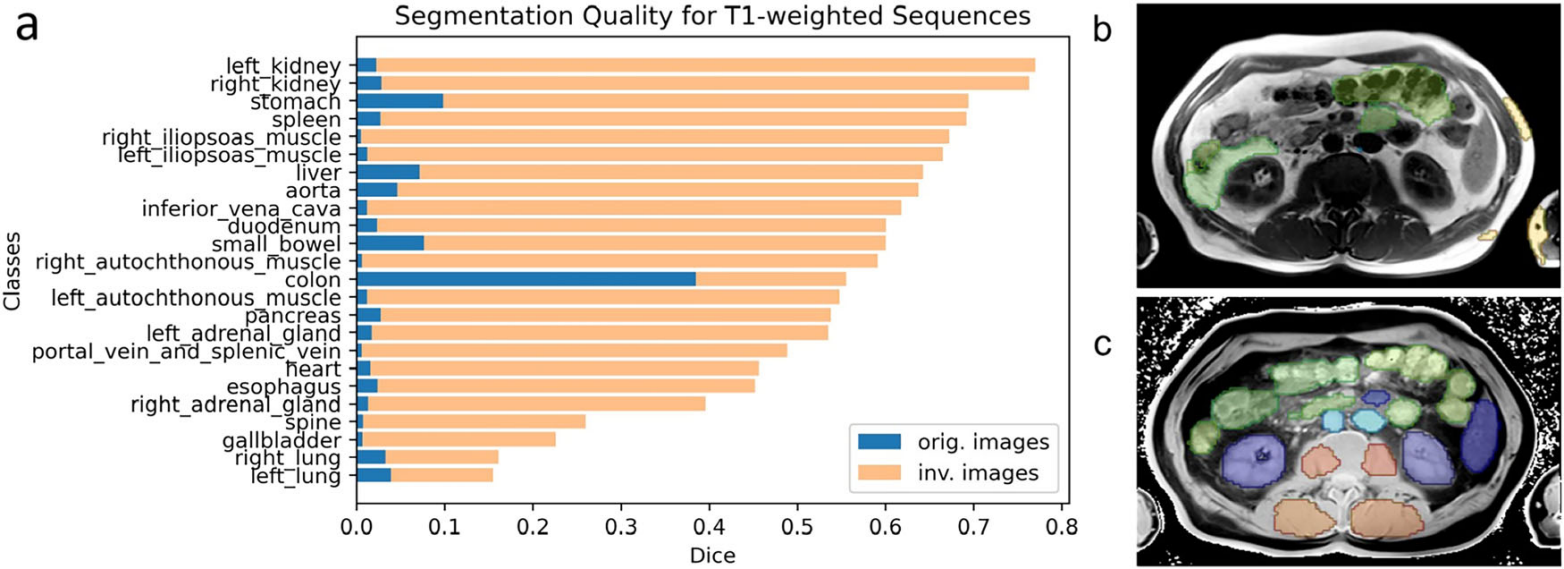
**a** Class-wise Dice similarity coefficient (DSC) of segmentations by TotalSegmentator-fast for T1w images. The figure shows the results for unprocessed images and inverted images with black background. The images on the right show a segmentation before (**b**) and after contrast inversion (**c**)

Inverting the contrast of images and setting the background values to zero improved segmentation quality for T1w images across all classes (Fig. 2); twenty classes had a DSC between 0.40 (right adrenal gland) up to 0.77 (right kidney). Although still improved, the worst segmentations were observed for the left and right lungs (DSC 0.15, 0.16), gallbladder (DSC 0.23), and vertebrae (DSC 0.26). The average DSC across all classes increased from 0.04 to 0.53 (Table 1). For the T2-weighted images, data augmentation led to a decrease in segmentation quality for all classes, except the aorta (DSC from 0.17 to 0.30). Plain inversion, without setting the background to zero, performed worse in all cases for both models compared to inversion with the background set to zero (Table 1). All results were statistically significant (*p* < 0.001, Table 2), except for the comparison between the inverted and inverted (black) preprocessing steps for tumor prediction in T2wfs sequences (*p* = 0.198). In this case, both methods failed to produce meaningful segmentations, leading to no significant difference between them.

**Table 1:** Dice similarity coefficient and 95% confidence intervals for TotalSegmentator

24 Classes (TotalSegmentator)			
Sequence	Unprocessed	Inverted	Inverted (black background)
T1	0.04 (0.01, 0.07)	0.28 (0.21, 0.34)	0.53 (0.46, 0.6)
T2fs	0.28 (0.21, 0.36)	0.06 (0.03, 0.08)	0.12 (0.09, 0.15)

*T1w* T1-weighted sequences, *T2wfs* T2-weighted fat-saturated sequences

**Table 2:** Pairwise comparisons of DSC between different preprocessing steps for T1w and T2wfs sequences

Comparison	24 Classes (TotalSegmentator)	Primary tumor (tumor model)
**T1**		
Unprocessed vs inverted	< 0.001	< 0.001
Unprocessed vs inverted (black)	< 0.001	< 0.001
Inverted vs inverted (black)	< 0.001	< 0.001
**T2wfs**		
Unprocessed vs inverted	< 0.001	< 0.001
Unprocessed vs inverted (black)	< 0.001	< 0.001
Inverted vs inverted (black)	< 0.001	0.198

For TotalSegmentator, the DSC was averaged across 24 classes, while for our tumor model, we used the DSC of primary tumor predictions. Statistical significance was assessed using Wilcoxon signed-rank tests, with all *p*-values adjusted using the Benjamini–Hochberg correction. All results were statistically significant, except for the comparison between the inverted and inverted (black) preprocessing steps for tumor prediction in T2wfs sequences. In this case, both methods failed to produce meaningful results, leading to no significant difference between them

*T1w* T1-weighted sequences, *T2wfs* T2-weighted fat-saturated sequences

To assess the performance of the renal tumor model, we focused on three distinct classes: tumor, adjacent-normal kidney (AN), and contralateral-normal kidney (CN). In an unprocessed T1w MRI, the model could not correctly segment any of the three classes (DSC ≤ 0.03). In unprocessed T2wfs sequences, it was able to roughly segment the kidneys with DSC values of 0.57 for AN and 0.63 for CN, while tumor segmentation remained unsuccessful (DSC: 0.12; *p* < 0.001) (Table 3). Adding the inversion step and setting the background to zero increased T1w segmentation accuracy for kidneys (DSC: 0.71 for AN; 0.76 for CN) and tumors (DSC: 0.42) but prevented segmentation in the T2wfs sequences (DSC ≤ 0.10 for all classes).

**Table 3:** Dice similarity coefficient and 95% confidence intervals for the primary tumor segmentations of the tumor model

Primary tumor (tumor model)			
Sequence	Unprocessed	Inverted	Inverted (black background)
T1w	0.04 (0.01, 0.07)	0.14 (0.09, 0.20)	0.42 (0.35, 0.49)
T2wfs	0.12 (0.07, 0.16)	0.01 (0.00, 0.02)	0.02 (0.00, 0.05)

*T1w* T1-weighted sequences, *T2wfs* T2-weighted fat-saturated sequences

For the specific case of T1w images with inverted contrast, tumors were correctly localized in 75 scans, incorrectly in 19 scans, and could not be detected in 6 scans. The tumors in these groups had a median volume of 35 cm^3^, 23 cm^3^, and 6 cm^3^, respectively. Tumors below the median volume of 29 cm^3^ were segmented with a DSC of 0.22, and tumors above the median volume with a DSC of 0.62. Spearman’s rank correlation showed a significant correlation between tumor volume and DSC (*p* < 0.001).

## Discussion

Creating MRI annotations from scratch is work-intensive and time-consuming. We showed that CT segmentation models paired with image inversion can create good pre-segmentations in MR images with DSCs up to 0.77. We demonstrated the feasibility for both a general multiclass and a specialized renal tumor model: using TotalSegmentator, we were able to create good pre-segmentations of abdominal organs in both T1w and T2wfs sequences. Further, we showed that a CT-trained renal tumor model can localize and segment large tumors in T1w MRI scans, if preceded by a contrast inversion step.

Whether to include the inversion step depends on the image modality. Both CT models were able to roughly segment abdominal organs in the T2wfs sequences with DSCs up to 0.63 (right kidney), without any preprocessing. Contrary, for the T1w sequences, the inversion step considerably increased segmentation quality. The difference is likely caused by the increased intensity of water in T2wfs images, where most organs appear brighter than the surrounding tissue. Of the four classes with a DSC below 0.4 in the T1w images, three (lungs, gallbladder) are filled with fluid or air, which could be the reason for the poor segmentation quality.

Renal tumor segmentation failed for T2wfs sequences but was successful in T1w sequences, when paired with the inversion step. Contrary to many abdominal organs with clear, distinctive borders, renal lesions can have similar attenuation to the kidneys [18]. Despite this, we were able to segment tumors in many instances. The segmentation quality greatly correlated with tumor volume, with larger tumors being localized and segmented much better. However, this could partially be a side-effect of using the DSC as a metric, which is more suitable for larger structures [19]. The results emphasize the importance of color gradients for the tested models. In particular, the contrast between participants’ bodies and the background air appears to be crucial. Consistently setting the background in an inverted image to black improved the segmentation of all classes, even for organs located in the center of the body.

We evaluated segmentation quality primarily in the context of abdominal structures. For musculoskeletal structures, improvements were demonstrated in the segmentation of autochthonous and iliopsoas muscles, as well as the spine, within T1w sequences (Fig. 2). While other bones were not explicitly tested, their inherent hyperdensity in CT and hypointensity across many MRI sequences suggest that the proposed approach may generalize to additional musculoskeletal structures. For nonmusculoskeletal regions, such as the brain, no supporting data were available to validate segmentation outcomes. However, it is noteworthy that MRI-specific segmentation methodologies for the brain are already well established in research settings [20], rendering a CT-to-MRI transfer approach potentially redundant for this anatomical region.

Previous studies have explored the transfer of annotations from CT to MRI images for downstream processing. Kieselmann et al [21] trained a 2D CycleGAN to generate synthetic MRI images from CT data. They subsequently trained a segmentation model exclusively on the synthetic images to segment the parotid glands, demonstrating that the model could effectively segment raw MRI as well. Similarly, Graf et al [22] evaluated various paired MRI-to-CT translation methods and showed that their synthetic CT images were sufficiently accurate for whole spine segmentation. However, machine learning-based image translation has significant limitations, including the potential for hallucination of artificial structures [23]. Furthermore, such models are often tailored to specific tasks and image modalities, limiting their generalizability to new problems. In contrast, our study does not rely on machine learning to create synthetic CT images. Instead, we propose the use of inversion techniques or, in the case of T2wfs sequences, avoiding augmentation altogether.

Ideally, our strategy should be used as the first step in an active learning framework, such as MONAI Label [24, 25], for annotating a new structure. Inversion with subsequent segmentation can be quickly applied to a large dataset. Annotators can then assess the quality at a glance and focus on the best segmentations, while saving the worst results for later. Given sufficient MRI sequences, it may even be enough to train an nnU-Net without any new or little annotation effort [26].

**Fig. 3:**
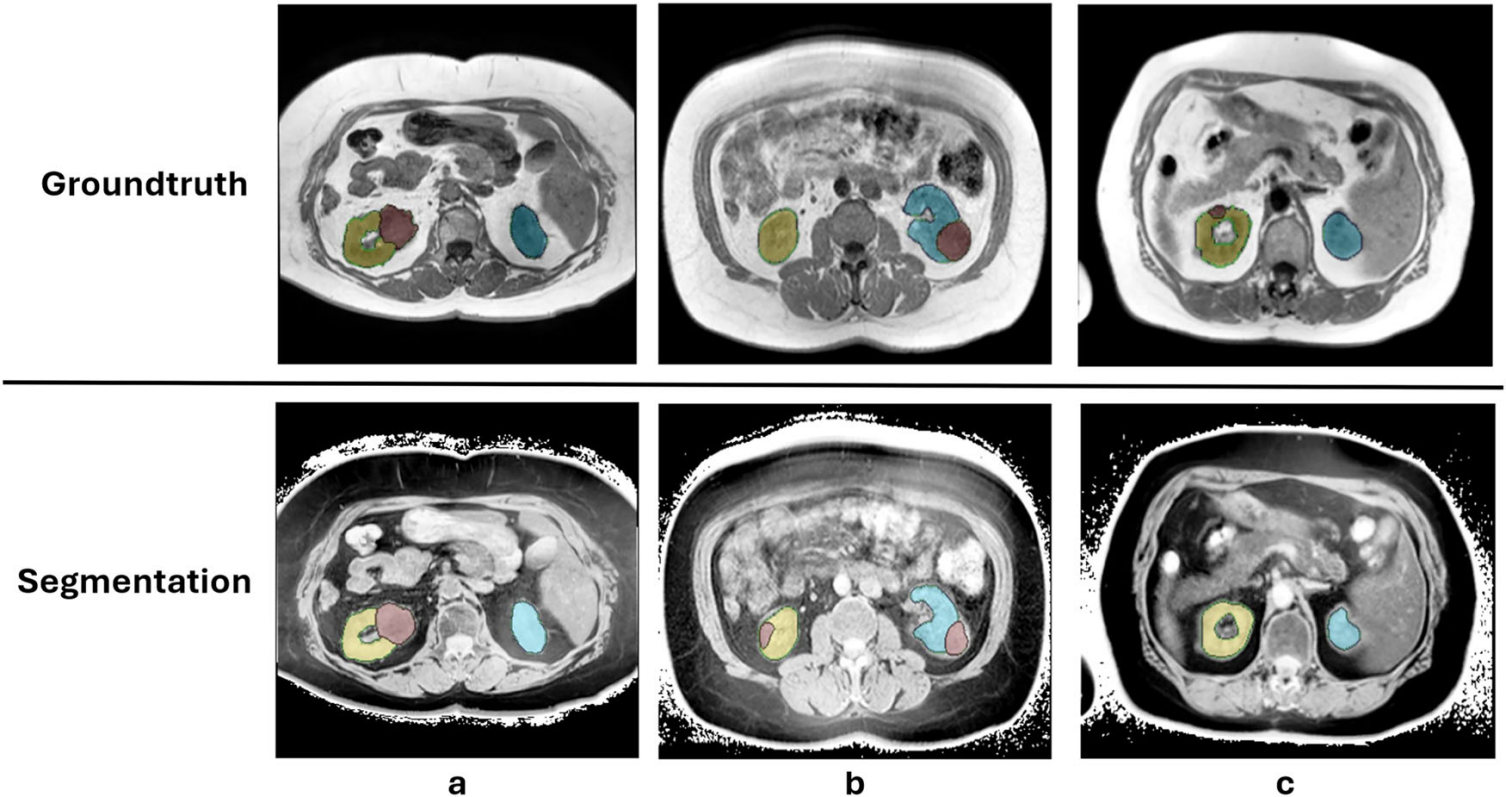
Reference standards and segmentations of kidneys and renal tumors in T1w sequences. **a** Ideal case: primary tumor and kidney are correctly separated (DSC 0.94). **b** The model segments a possible secondary tumor that is not included in the reference standard, resulting in a bad evaluation (DSC 0.51). **c** Model fails to correctly delineate the tumor (DSC 0.00)

Our study has limitations. First, we created our reference standard using both manual annotation and automatic segmentation. Doing this reduces the meaningfulness of the DSC, as we cannot assume the reference standard to be completely correct. However, we consider it sufficient in the context of our study, as the goal is not 100% accuracy, but rather creating pre-segmentations for a faster annotation process. Second, we focused on primary renal tumors and disregarded secondary tumors or metastasis. A segmentation of these would have been classified as incorrect by our evaluation pipeline, while a radiologist might have decided otherwise (Fig. 3). Finally, our approach was evaluated on a retrospective dataset obtained from a single institution. Given the substantial variability in MRI acquisition parameters across scanners and institutions, the generalizability of the method beyond this dataset remains uncertain. A true assessment of its accuracy would require external validation across multiple centers. Future work may explore this to further assess and refine the method’s applicability.

Our results underline that transferring available CT models, including models of pathologic lesions, to the MRI domain can provide a viable efficiency boost in projects requiring large-scale pixel-wise annotation. More sophisticated modality-transfer methods could potentially increase generalizability, however, implementing these complex models can be challenging and may be disproportionate for small-scale projects. For certain sequences, CT models and image inversion alone can be sufficient to achieve satisfactory results.

## Data Availability

A Python implementation of the inversion step is available on https://github.com/hhaentze/Annotation-Acceleration. The data that support the findings of this study are available from *Charité—Universitätsmedizin Berlin*, but restrictions apply to the availability of these data, which were used under license for the current study, and so are not publicly available. Data are, however, available from the authors upon reasonable request and with permission of *Charité—Universitätsmedizin Berlin*.

## Abbreviations

CT: Computed tomography
DSC: Dice similarity coefficient
MRI: Magnetic resonance imaging
T1w: T1-weighted
T2wfs: T2-weighted fat-saturated
